# Use of Telehealth for Domiciliary Follow-up After Hematopoietic Cell Transplantation During the COVID-19 Pandemic: Prospective Pilot Study

**DOI:** 10.2196/26121

**Published:** 2021-03-12

**Authors:** Alberto Mussetti, Maria Queralt Salas, Maria Condom, Maite Antonio, Cristian Ochoa, Iulia Ivan, David Jimenez Ruiz-De la Torre, Gabriela Sanz Linares, Belen Ansoleaga, Beatriz Patiño-Gutierrez, Laura Jimenez-Prat, Rocio Parody, Ana Sureda-Balari

**Affiliations:** 1 Clinical Hematology Department Institut Català d'Oncologia-Hospitalet L'Hospitalet de Llobregat, Barcelona Spain; 2 Institut d'Investigació Biomèdica de Bellvitge Barcelona Spain; 3 Oncohematogeriatrics Unit Institut Català d'Oncologia-Hospitalet Barcelona Spain; 4 Psycho-Oncology Unit, Institut Català d’Oncologia ICOnnecta’t Health Program Hospital Duran i Reynals L’Hospitalet de Llobregat, Barcelona Spain; 5 Clinical Psychology and Psychobiology Department Faculty of Psychology University of Barcelona Barcelona Spain

**Keywords:** SARS-CoV-2, COVID-19, hematology, hematopoietic cell transplantation, telemedicine, mortality, surveillance, monitoring, stem cell, transplant, app, medical device

## Abstract

**Background:**

Patients who have recently received a hematopoietic cell transplant (HCT) are at higher risk of acute complications in the first weeks after discharge, especially during the COVID-19 pandemic.

**Objective:**

The aim of this study was to test the use of a telehealth platform for the follow-up of HCT patients during the first two weeks after discharge.

**Methods:**

In total, 21 patients who received autologous or allogeneic HCT for hematological malignancies were screened from April 30, 2020, to July 15, 2020. The telehealth platform assisted in the daily collection of vital signs as well as physical and psychological symptoms for two weeks after hospital discharge. The required medical devices (oximeter and blood pressure monitor) were given to patients and a dedicated smartphone app was developed to collect this data. The data were reviewed daily through web-based software by a hematologist specializing in HCT.

**Results:**

Only 12 of 21 patients were able to join and complete the study. Technological barriers were the most frequent limiting factor in this study. Among the 12 patients who completed the study, adherence to data reporting was high. The patients’ experience of using such a system was considered good. In two cases, the system enabled the early recognition of acute complications.

**Conclusions:**

This pilot study showed that telehealth systems can be applied in the early posttransplant setting, with evident advantages for physicians and patients for both medical and psychological aspects. Technological issues still represent a challenge for the applicability of such a system, especially for older adult patients. Easier-to-use technologies could help to expand the use of telehealth systems in this setting in the future.

## Introduction

Patients receiving a hematopoietic cell transplant (HCT) have a high risk of developing severe acute toxicities in the early posttransplant period. An increased mortality risk has been observed in HCT patients who go on to develop COVID-19, with an estimated mortality rate between 20%-40% [1,2]. Although other hematological procedures can be postponed in case of emergency, there is an international agreement to not defer transplant procedures, especially for rapidly progressing diseases such as acute leukemias and aggressive lymphomas. Due to this, it is necessary to improve the domiciliary clinical monitoring of patients who received transplants to rapidly detect clinical deterioration. In addition, unnecessary in-person visits that might increase the risk of intrahospital contagion should be reduced. During the last few years, smart devices for assessing vital signs or physical activity have emerged as breakthrough innovations in the oncological setting [3-5]. Digital technologies allow clinicians to perform real-time monitoring of a patient’s clinical status. In patients with COVID-19, the use of devices such as digital oximeters allow for early detection of clinical deterioration and a safer domiciliary follow-up. This is of paramount importance for patients who have received transplants, who have higher COVID-19–related mortality than the general population.

## Methods

The aim of this study (SMARTCOVID19 study) is to report the feasibility of a real-time patient monitoring system through the use of a smartphone app and mobile health care devices. The institutional review board of Institut Catalá d'Oncologia - Hospitalet approved the study. Inclusion criteria were the following: those aged >18 years who had received autologous or allogeneic HCT while hospitalized and have a smartphone with an operating system able to run the SMARTCOVID19 app. Those who did not have adequate social support were excluded from the study.

Patient education regarding the use of the platform was provided by a hematologist at the time of enrollment, which was 1-2 days before hospital discharge.

Vital signs (heart rate, oxygen saturation, and arterial blood pressure) were collected daily using clinically validated oximeters (Onyx II, Nonin Inc) and a blood pressure monitor (iHealth Track, iHealth Labs), while temperature was measured using domiciliary thermometers. Patients were educated on how to measure their respiratory frequency. A checklist of clinical symptoms was completed daily (presence of cough, myalgia, headache, fatigue, dyspnea, emesis, odynophagia, rhinitis, conjunctivitis, and chest pain). An analog visual scale “thermometer” (0-10) used to detect potential cases of anxiety or depressive disorders was completed by patients daily. Scores of >6 resulted in an automatic referral to a psycho-oncologist through the platform, who would contact the patient and evaluate whether psychological support was needed via videoconference. A chat service was available for nonurgent communications. All data were reported to an online platform through a smartphone app (“Saludencasa,” Fundación Trilema) compatible with Apple (iOS Version 9 or higher) and Android systems (Version 6 or higher). A hematologist with experience with HCTs reviewed all patient data daily. Programmed alarms were set in the event of any of the following situations: fever >38 C, oxygen saturation <92%, tachycardia >125 beats per minute, hypotension (systolic blood pressure <90 mm Hg, diastolic blood pressure <60 mm Hg), altered mental status, and persistent emesis or diarrhea (lasting more than 48 hours). In case of alarm activation, the hematologist contacted the patient by phone to evaluate the need for an in-person visit and determine the clinical management steps that were considered most appropriate. Outpatient monitoring started from the day of hospital discharge 2 days and continued for 14 days. The study accrual period was April 30, 2020, to July 15, 2020. Data were collected prospectively and all patients signed informed consent forms. Finally, two weeks after the end of the recruitment period, patients were contacted by phone and asked to answer a satisfaction questionnaire.

## Results

During the study period, 16 of 21 patients who received transplants were successfully recruited into the study (76% feasibility). Reasons for patients not being enrolled were the following: language incompatibility (1 patient), no consent (1 patient), and no compatible smartphone (3 patients). Of the 16 enrolled patients, the median age was 50 years (range 22-70 years), 38% (n=6) were female, and 94% (n=15) had lymphatic diseases. In addition, 38% (n=6) of HCTs were autologous and 62% (n=19) were allogeneic.

Of the 16 enrolled patients, 4 were not able to use the app due to an inability to use smartphone apps in general. Of the remaining 12 patients, average adherence to reporting study data (median number of days reported across all patients during the planned 14-day study period) was as follows: 89% for temperature, 90% for oxygen saturation, 70% for respiratory frequency, 85% for cardiac frequency, 89% for blood pressure, 65% for symptoms reporting, and 71% for emotional distress.

Automatic alarms were activated only 3 times: twice for the presence of clinical symptoms and once for emotional distress. Only one patient spoke with the psycho-oncologist via videoconference. In total, 4 patients used the chat service to communicate with hospital personnel.

Despite the feasibility nature of the study, data collected with the digital system helped the clinician to recognize calcineurin inhibitor–induced arterial hypertension in one patient and acute cutaneous graft-versus-host disease (grade I) in another patient.

Only two patients in this cohort were readmitted within 14 days of discharge, both due to grade 4 odynophagia related to herpes simplex virus 1/2 reactivation.

The patients’ responses to the survey questions about their experience with the telehealth system are reported in Table 1.

**Table 1:** Patients’ responses to survey questions about their experience with the telehealth system (n=12).

Question	Mean score (scored from 1-5, where 1=disagree and 5=agree)
Overall satisfaction with the telehealth system	4.67
Did you feel safer at home with the use of the telehealth system?	4.67
Do you think that using such a device has improved your domiciliary follow-up?	4.67
Was the app easy to use?	4.50
When you feel well, would you be comfortable substituting in-person visits with telemedicine?	3.50

## Discussion

Our prospective study showed that the use of mobile health care devices and smartphone apps for self-reported outcomes is feasible in the post-HCT setting. In a study conducted by Nawas et al [6], it was found that telehealth evaluations could be useful in the early peritransplant period, with a high satisfaction rate among patients. In our study, we found that patients felt safer when using the telehealth system. However, only a few patients would completely substitute an in-person visit with telehealth monitoring. This suggests that an in-person visit with a medical doctor is still the preferred follow-up modality for patients who have received transplants. Apart from vital signs monitoring and blood test results, which could be evaluated without the patient present, other aspects of medical care cannot be replaced by telehealth. Human contact and empathy are still largely needed during a visit. With respect to feasibility and adherence, two observations emerged from our study. The first is that only 57% of the potential patients used the telehealth system in the end. Technological barriers such as incompatible smartphones or inexperience in using smartphone apps were the main causes for study failure. This problem was more frequently observed in older adult patients living alone or without caregivers able to help them use the devices. A solution to this could be to automate data collection through the use of devices that automatically monitor and report this data. Newer wearable devices could allow for real-time monitoring of patients’ heart rate, oxygen saturation, and physical activity in an automated manner. A second technological issue became apparent when evaluating the reporting adherence of the people who succeeded in using the platform. In this case, adherence to reporting clinical parameters such as respiratory frequency or symptoms was low. The use of currently available wearable devices could also resolve this adherence issue. In addition to technical issues, it is possible that psychological barriers could have contributed to reducing study adherence. Better patient education on how to use this system could also improve adherence, and should always be considered when applying digital medicine. Finally, the quality of telehealth monitoring should be complemented and improved by the collection of other clinically relevant parameters. For example, a virtual physical examination could be carried out through the use of high-quality video calls, digital stethoscopes, and weight scales [6,7]. In our study, the system worked well for detecting acute complications such as infections and dehydration. It is useful for monitoring the early posttransplant period, when acute complications are more frequent. For long-term follow-up, this system should be implemented with other technologies (eg, ones that allow visual communication between physicians and patients).

A study limitation was the small number of patients that completed the study. However, the study population was considered sufficient for a pilot study. In fact, the results obtained should be used to improve the design of the next study, which will recruit a larger number of patients. In addition, the uptake and use of newer technologies could be influenced by country or hospital resources. Such a system would not be feasible to implement within hospitals or health care systems that cannot afford such expenses. This applicability issue is common to the majority of studies using newer technologies.

In conclusion, telehealth monitoring could potentially improve patient follow-up in terms of both physical and psychological outcomes. This is especially true whenever an external cause (such as the COVID-19 pandemic) impedes in-person visits. Technological issues still represent a barrier to the wider application of telehealth monitoring systems in a medical setting and these issues should be considered for future studies.

## Abbreviations

HCT: hematopoietic cell transplant

## References

[1] Shah G, DeWolf S, Lee Y, Tamari R, Dahi P, Lavery J, Ruiz J, Devlin SM, Cho C, Peled JU, Politikos I, Scordo M, Babady NE, Jain T, Vardhana S, Daniyan A, Sauter CS, Barker JN, Giralt SA, Goss C, Maslak P, Hohl TM, Kamboj M, Ramanathan L, van den Brink MR, Papadopoulos E, Papanicolaou G, Perales MA (2020). Favorable outcomes of COVID-19 in recipients of hematopoietic cell transplantation. J Clin Invest.

[2] Passamonti F, Cattaneo C, Arcaini L, Bruna R, Cavo M, Merli F, Angelucci E, Krampera M, Cairoli R, Della Porta MG, Fracchiolla N, Ladetto M, Gambacorti Passerini C, Salvini M, Marchetti M, Lemoli R, Molteni A, Busca A, Cuneo A, Romano A, Giuliani N, Galimberti S, Corso A, Morotti A, Falini B, Billio A, Gherlinzoni F, Visani G, Tisi MC, Tafuri A, Tosi P, Lanza F, Massaia M, Turrini M, Ferrara F, Gurrieri C, Vallisa D, Martelli M, Derenzini E, Guarini A, Conconi A, Cuccaro A, Cudillo L, Russo D, Ciambelli F, Scattolin AM, Luppi M, Selleri C, Ortu La Barbera E, Ferrandina C, Di Renzo N, Olivieri A, Bocchia M, Gentile M, Marchesi F, Musto P, Federici AB, Candoni A, Venditti A, Fava C, Pinto A, Galieni P, Rigacci L, Armiento D, Pane F, Oberti M, Zappasodi P, Visco C, Franchi M, Grossi PA, Bertù L, Corrao G, Pagano L, Corradini P, ITA-HEMA-COV Investigators (2020). Clinical characteristics and risk factors associated with COVID-19 severity in patients with haematological malignancies in Italy: a retrospective, multicentre, cohort study. Lancet Haematol.

[3] Patel M, Cooney MM, Caimi PF, Randall RE, Metheny LL, Mendiratta P, Bruno DS, Silverman P (2020). Pilot study for the use of wearable devices for patients undergoing oral chemotherapy. JCO.

[4] Liao Y, Thompson C, Peterson S, Mandrola J, Beg MS (2019). The Future of Wearable Technologies and Remote Monitoring in Health Care. American Society of Clinical Oncology Educational Book.

[5] Cox SM, Lane A, Volchenboum SL (2018). Use of Wearable, Mobile, and Sensor Technology in Cancer Clinical Trials. JCO Clinical Cancer Informatics.

[6] Nawas MT, Landau HJ, Sauter CS, Featherstone CA, Kenny SA, Rodriguez ES, Johnson LG, Giralt SA, Scordo M (2020). Pilot Study of Telehealth Evaluations in Patients Undergoing Hematopoietic Cell Transplantation. Biol Blood Marrow Transplant.

[7] Lupo-Stanghellini MT, Messina C, Marktel S, Carrabba MG, Peccatori J, Corti C, Ciceri F (2020). Following-up allogeneic transplantation recipients during the COVID-19 pandemic. Lancet Haematol.

